# Calciphylaxis: Beating the Odds

**DOI:** 10.7759/cureus.76996

**Published:** 2025-01-06

**Authors:** José Mário Bastos, Joana Medeiros, Johanna Viana, Catarina Oliveira Silva, Maria Joao Rocha

**Affiliations:** 1 Nephrology, Unidade Local de Saúde de Braga, Braga, PRT; 2 Nephrology, Unidade Local de Saúde de Matosinhos, Porto, PRT

**Keywords:** calciphylaxis, chronic kidney disease, hemodialysis, partial-thickness skin grafting, sodium thiosulfate

## Abstract

Calciphylaxis is an obliterative vasculopathy characterized by ischemic cutaneous necrosis secondary to the calcification of small- and medium-caliber blood vessels. This condition poses a significant therapeutic challenge and is often associated with poor outcomes.

We report the case of a 59-year-old man who had been on renal replacement therapy for 28 years and presented with a painful necrotic ulcer on his left leg, which progressively worsened over three weeks. A skin biopsy confirmed the diagnosis of calciphylaxis. The treatment approach included intensified hemodialysis, optimization of mineral and bone disorder management, treatment of secondary bacterial infection, intravenous sodium thiosulfate infusion, chemical and surgical debridement, and partial-thickness skin grafting. This comprehensive strategy resulted in complete wound healing within three months.

This case highlights the importance of early diagnosis and a multidisciplinary approach in managing calciphylaxis, a rare condition associated with a highly unfavorable prognosis.

## Introduction

Calciphylaxis, also known as calcific uremic arteriolopathy, is a rare and life-threatening obliterative vasculopathy characterized by ischemic skin necrosis due to the calcification and occlusion of small- and medium-sized blood vessels. This vascular calcification leads to luminal narrowing and thrombosis, resulting in reduced blood flow, tissue ischemia, and subsequent skin necrosis [1]. Although it is most commonly observed in patients with end-stage renal disease (ESRD) undergoing dialysis, calciphylaxis can also occur in earlier stages of chronic kidney disease (CKD), after kidney transplantation, and, rarely, in individuals with normal kidney function [1,2]. The condition is associated with a poor prognosis, with a one-year mortality rate reaching up to 80% in patients with CKD [3].

Numerous risk factors have been associated with calciphylaxis, including disturbances in calcium-phosphate metabolism, obesity, diabetes mellitus, female sex, white race, vitamin K deficiency, and prolonged use of calcium-based phosphate binders or vitamin D supplements [1,3]. Additionally, patients often present with elevated parathyroid hormone (PTH) levels, hyperphosphatemia, and a deficiency of natural inhibitors of calcification, such as fetuin-A and matrix Gla protein [2, 4]. However, not all affected individuals display these abnormalities, highlighting the multifactorial and poorly understood pathogenesis of this condition [4].

The clinical presentation is typically characterized by painful, ischemic skin lesions that frequently progress to necrosis, increasing the risk of infection and sepsis [3]. Given its dismal prognosis and limited therapeutic options, calciphylaxis underscores the critical need for early recognition and a multidisciplinary approach to treatment [4].

## Case presentation

A 57-year-old male presented to the emergency department with a three-week history of a painful ulcer that appeared spontaneously on the back of his left lower leg and was progressively increasing in size. He denied fever, chills, or any other systemic symptoms.

The patient had a 28-year history of ESRD, managed with two kidney transplants that lasted a total of 11 years, along with 17 years of hemodialysis, including periods between the two transplants and following the second transplant. His medical history was notable for secondary hyperparathyroidism, treated with etelcalcetide (5 mg three times per week) and alfacalcidol (0.5 mcg three times per week), as well as paroxysmal atrial fibrillation managed with long-term anticoagulation using warfarin.

**Figure 1:**
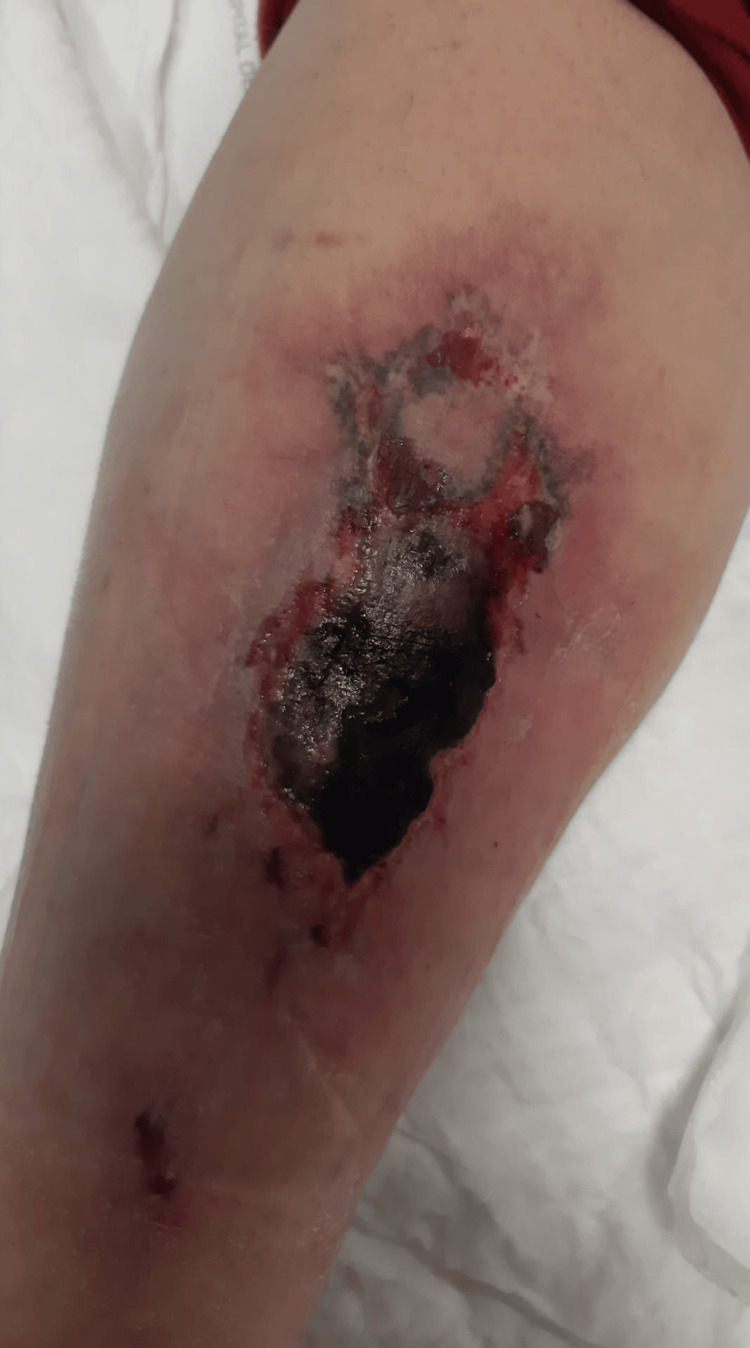
Necrotic lesion at admission. Necrotic ulcer approximately 4 cm in size on the posterior left lower leg, with a central black eschar and an erythematous border, consistent with ischemic necrosis and calciphylaxis.

On physical examination, the patient was afebrile, with a blood pressure of 135/77 mmHg and a heart rate of 75 beats per minute. He presented with a necrotic ulcer approximately 4 cm in diameter on the back of his left lower leg (Figure 1). There were no signs of cellulitis or systemic infection at the time of presentation. Cardiopulmonary and abdominal examinations were unremarkable.

Laboratory results at admission are summarized in Table 1.

**Table 1:** Laboratory results at admission. *For patients with chronic kidney disease (CKD) on hemodialysis, the target PTH range is approximately 2-9 times the upper limit of the normal range (ULN). RR, reference range

Parameter	Result	Units	RR
Parathyroid hormone (PTH)	393.58	pg/mL	10-65*
Serum calcium	9.2	mg/dL	8.3-10.6
Phosphate	4.0	mg/dL	2.4-5.1
C-reactive protein (CRP)	3.5	mg/L	<5.0

Given the clinical presentation and the patient’s history, calciphylaxis was suspected, and the patient was admitted under the care of the nephrology team. A skin biopsy confirmed the diagnosis, revealing vascular calcification and thrombosis in small- and medium-sized arterioles, accompanied by tissue necrosis. Non-calcium-based phosphate binders (sevelamer) were initiated, and the calcimimetic therapy (etelcalcetide) was continued. Hemodialysis was intensified to four sessions per week to optimize metabolic control and fluid balance. Warfarin was replaced with enoxaparin adjusted to renal function, and alfacalcidol was discontinued.

On the fifth day of hospitalization, the patient developed signs of local infection around the ulcer, including erythema, warmth, and swelling. Laboratory tests showed a leukocytosis of 13,600/μL with 10,200 neutrophils/μL and a C-reactive protein (CRP) of 115 mg/L. Empiric antibiotic therapy with vancomycin and meropenem was initiated to target possible Gram-positive and Gram-negative pathogens. Blood cultures and a swab of the lesion were collected but yielded no microbial growth. Antibiotic therapy was continued for 14 days, leading to the resolution of the inflammatory signs.

Intravenous sodium thiosulfate therapy was started on the seventh day of hospitalization, at a dose of 25 grams three times weekly after dialysis. Early involvement of the Plastic Surgery team facilitated both chemical debridement with silver sulfadiazine and surgical debridement to remove necrotic tissue and promote wound healing. Although the patient was listed for hyperbaric oxygen therapy, the wound began to show significant improvement before this intervention could be initiated.

**Figure 2:**
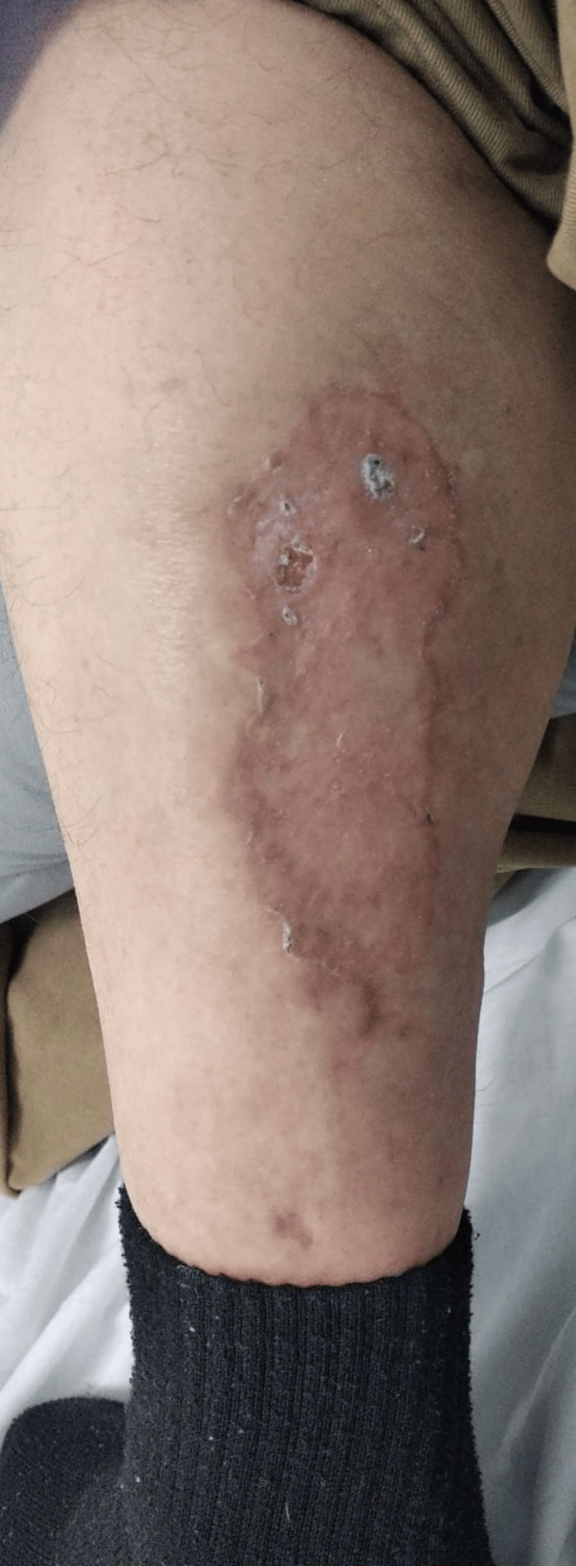
Calciphylaxis lesion at the one-month follow-up consultation. Healed lesion on the posterior left lower leg at the one-month follow-up, showing complete wound closure with residual scarring and no evidence of active infection or recurrence.

By the 41st day of hospitalization, a partial-thickness skin graft was harvested from the ipsilateral thigh and successfully applied to the wound bed. The patient continued to demonstrate gradual improvement, achieving complete wound healing and being discharged on the 47th day of hospitalization. At the one-month outpatient follow-up consultation, the lesion was fully healed, with no signs of recurrence or complications (Figure 2).

## Discussion

Calciphylaxis, or calcific uremic arteriolopathy, is a rare but life-threatening condition that primarily affects patients with ESRD undergoing dialysis. Despite advancements in understanding the disease, calciphylaxis remains associated with a poor prognosis, with mortality rates reported to range between 50% and 80% within the first year, largely due to complications such as sepsis and delayed wound healing [5,6]. Furthermore, calciphylaxis carries a significant risk of recurrence, particularly in patients with ongoing inflammation or inadequate control of underlying risk factors, such as hyperparathyroidism and elevated calcium-phosphorus product levels [7].

This case underscores the importance of identifying risk factors and implementing early interventions in patients at high risk for calciphylaxis. The patient’s long-standing ESRD, having been on renal replacement therapy (RRT) for 28 years, coupled with secondary hyperparathyroidism and chronic warfarin use, placed him in a high-risk category. Warfarin is a known contributor to calciphylaxis due to its inhibition of vitamin K-dependent proteins, such as matrix Gla protein, a critical inhibitor of vascular calcification. Its replacement with enoxaparin may have mitigated further progression of vascular calcification. The lower extremities, particularly the legs, are common sites for calciphylaxis lesions due to reduced vascular reserve, ischemic predisposition, and increased susceptibility to chronic microtrauma, all of which exacerbate tissue necrosis in these regions [5-7].

Management of calciphylaxis requires a multidisciplinary approach addressing both systemic and local factors contributing to disease progression. Sodium thiosulfate was initiated as part of the therapeutic regimen. Although its precise mechanism of action is not fully understood, sodium thiosulfate is thought to reduce vascular calcification by chelating calcium, enhancing its solubility, and mitigating oxidative stress. It has also been associated with improvements in pain and wound healing in patients with calciphylaxis [8,9].

Optimization of mineral and bone metabolism was another cornerstone of this patient’s management. Sevelamer was introduced to reduce phosphorus while minimizing further calcium overload. Since the calcium-phosphorus product is one of the main risk factors for the onset and progression of calciphylaxis, it was used even with normal serum phosphorus levels, effectively reducing the product without ever causing hypophosphatemia. Etelcalcetide was continued to address secondary hyperparathyroidism, without raising the phosphate or calcium levels. These interventions are essential in controlling the calcium-phosphate balance and preventing vascular calcification and subsequent tissue necrosis [5,10].

Secondary bacterial infection of necrotic ulcers is a common and serious complication of calciphylaxis [11]. In this case, the development of local signs of infection, including erythema, swelling, and elevated inflammatory markers, prompted the timely initiation of broad-spectrum antibiotics. The absence of microbial growth in blood cultures and lesion swabs highlights the challenge of identifying causative organisms in such cases [6,11]. Nonetheless, early and effective antimicrobial treatment was crucial in resolving the infection and preventing systemic complications.

The involvement of the Plastic Surgery team was decisive in achieving a successful outcome. Early debridement, both chemical and surgical, was essential to create an optimal environment for wound healing. Chemical debridement with silver sulfadiazine not only controlled bacterial colonization but also facilitated autolytic debridement by maintaining a moist wound bed. Surgical debridement further ensured the removal of nonviable tissue. A partial-thickness skin graft, harvested from the ipsilateral thigh, was successfully applied to the prepared wound bed. This intervention accelerated wound closure and minimized the risk of long-term complications such as chronic infection or limb dysfunction. The early collaboration with plastic surgery points to the importance of integrating specialized surgical care into the management of calciphylaxis, particularly for large or refractory wounds [5,12].

## Conclusions

This case highlights the role of early recognition and a multidisciplinary approach in the management of calciphylaxis, a condition that poses significant therapeutic challenges. A combination of systemic therapy, advanced wound care, and surgical interventions was key to achieving a positive outcome in this patient. Continued research is necessary to enhance treatment protocols and improve survival rates for those affected by this rare but severe disease.

With a treatment plan guided by the latest literature and a close collaboration with the plastic surgery team, complete therapeutic success was achieved. This case underscores the importance of maintaining a high index of suspicion and providing timely interventions for a disease that, although rare, is frequently associated with poor outcomes.
